# Perspectives on healthcare, chronic non-communicable disease, and healthworlds in an urban and rural setting

**DOI:** 10.3402/gha.v7.25317

**Published:** 2014-09-24

**Authors:** Daniel Lopes Ibanez-Gonzalez

**Affiliations:** MRC/Wits Developmental Pathways for Health Research Unit, School of Clinical Medicine, Faculty of Health Sciences, University of the Witwatersrand, Johannesburg, South Africa

**Keywords:** Habermas, chronic non-communicable disease, healthworld, comparative case study, South Africa

## Abstract

**Background:**

Amidst diverging discourses describing chronic non-communicable disease (NCD) and healthcare access, the hermeneutical tradition within sociology, particularly as exemplified in the work of Jurgen Habermas, provides a starting point for exploring and interpreting the experiences of chronic illness and healthcare access. In this study, we aimed to understand how women living with NCDs experience their illness and access healthcare in an urban and rural context.

**Methods:**

This study was a mixed-methods comparative case study of the healthcare access experiences of women with NCDs in an urban and rural area in South Africa. The core of the study methodology was a comparative qualitative case study, with quantitative methods serving to contextualise the findings.

**Results:**

The cross-sectional survey describes a low resource population with a high prevalence of NCDs. Slightly over half the respondents in urban Soweto (50.7%) reported having at least one NCD. Only around a third (33.3%) of these participants reported accessing formal healthcare services in the past 6 months. Similar trends were found in the review of research carried out in rural Agincourt. The qualitative case study in Soweto is characterised by a preoccupation with how medicine from the clinic interacts with the body. The Agincourt qualitative case study highlights the importance of church membership, particularly of African Christian Churches, as the strongest factor motivating against the open use of traditional medicine.

**Discussion:**

A consideration of the findings suggests five broad themes for further research: 1) processes of constructing body narratives; 2) encounters with purposive–rational systems; 3) encounters with traditional medicine; 4) encounters with contemporary informal medicine; and 5) religion and healthcare. These five themes constitute the beginning of a comprehensive schema of the lifeworld/healthworld.

South Africa is undergoing a health transition characterised by the simultaneous occurrence of chronic and infectious diseases (1). Evidence from South Africa suggests that the increasing trend in chronic non-communicable disease (NCD) prevalence coexists with low healthcare utilisation (2). This has been attributed to the poor affordability and availability of healthcare services in South Africa (3), and a healthcare system still marked by apartheid-era inefficiencies (4). Although a great deal of progress has been made with regard to South Africa’s health system reforms (1, 4), the full achievement of equitable distribution of healthcare through a primary healthcare (PHC) approach has been hampered by a number of obstacles, including health worker shortages, inequities in resource distribution, and shortcomings in healthcare leadership (5).

Approaches to addressing increasing NCD prevalence are of two types:

1. The ‘objectivist’ or ‘positivist’ approach is characterised by a heavy reliance on quantitative measures of biomedical definitions of disease, which conceive ‘disease’ as some deviation from a biomedical norm (6).
2. The ‘hermeneutic’ or ‘social constructionist approach’ is characterised by an interest in the experience of ‘illness’ and the interpretive work performed by people with ‘chronic illness’ (6).

A social constructionist approach is rooted in the conceptual distinction between ‘illness’, understood as the lived experience of disease, and ‘disease’, understood as the biological condition or the reinterpretation of the illness experience by specialists in terms of technical classifications (7, 8). Drawing from both paradigms, this paper refers to ‘NCDs’ when engaging with biomedical and public health literature and to ‘chronic illness’ when describing the illness experiences of the research participants.

The present study is located in a context marked by multiple transitions: the transition from high prevalences of infectious to chronic diseases, the transition of a racially fragmented and inefficient healthcare system to an integrated healthcare system, and the transition of individual healthcare practices from informal to formal healthcare treatment. Given the high prevalence of NCDs as well as the dynamic nature of healthcare systems in South Africa, the key question for this study was how the diverse concepts within objectivist and social constructionist public healthcare approaches could be used to contribute to our understanding of NCDs and healthcare access.

## Introducing the lifeworld/healthworld

The hermeneutic tradition within sociology, particularly as exemplified in the work of Jurgen Habermas, provides a starting point for exploring and interpreting narratives of chronic illness and healthcare access. It is within this tradition that researchers of chronic illness and healthcare workers have been called upon to reflexively pursue the enhanced rationalisation of the lifeworld (9). The lifeworld may be understood as that culturally transmitted and linguistically organised stock of background knowledge which a person brings to their daily communicative encounters with others and with institutions (10). The concept of the ‘healthworld’ draws on the same hermeneutic tradition. It is a description of the complex of health beliefs and behaviours of individuals in relation to the ailing body (11).

## Study aim and objectives

The broader vision behind the study was to formulate a schematic framework of the lifeworld/healthworld and to explore its implications for public health research and policy around NCDs. Given this goal, the aim of the study was to understand how women with NCDs experience their illness and access healthcare in an urban and rural context. Specific objectives included:

1. Formulating a historical–comparative community description of the study sites using primary and secondary quantitative data
2. Analysing the experiences of chronic illness and healthcare access in an urban and a rural area

## Methodology

This study is a mixed-methods comparative case study of the healthcare access experiences of women with NCDs in an urban and rural area in South Africa. The core of the study methodology is a comparative qualitative case study, with quantitative methods serving to contextualise the findings.

### The study sites

The urban component of the study was conducted in Birth to Twenty (Bt20), a birth cohort study located in Soweto, Johannesburg which enrolled all singleton children born to women resident in the area from April to June 1990. From its inception, Bt20 has conducted multidisciplinary research in tracking the growth, health, wellbeing, and educational progress of urban children throughout their lives in Soweto, Johannesburg (12).

The rural component of the study was conducted in Agincourt, a subdistrict of the Bushbuckridge district in Mpumalanga province, situated in the rural north-east of South Africa near the Mozambican border. Agincourt was established as a district health demonstration site in 1992 by the University of Witwatersrand’s Health Systems Development Unit and the then ‘homeland’ health service (13, 14).

### Describing the context of the case studies

#### Urban context (part 1): secondary data analysis

Secondary data collected by Bt20 was used to construct a historical overview of the use of formal and informal healthcare services in Soweto. These variables constitute composite scores based on measures and types of healthcare utilisation asked of the Bt20 caregivers in various successive waves of data collection.

#### Urban context (part2): Soweto quantitative survey

A large scale survey was conducted in Soweto to contextualise the findings of the qualitative case study. The survey was a cross-sectional study of the primary caregivers (female heads of households) of the Bt20 cohort. A semi-structured questionnaire was administered at home by a team of research assistants in the home language of the study participants (mainly *seSotho* and *isiZulu*) between November 2008 and June 2010.

The questionnaire included a number of domains. The demographic section included demographic measures, asset-based socio-economic status, employment status, and religious affiliation and adherence. The general healthcare access section included measures of availability and affordability of healthcare services, medical aid, and perceived obstacles to accessing public healthcare. The specific healthcare access section included measures of recent illness and healthcare services accessed, as well as experiences of the healthcare visit. The health-seeking behaviour section included measures of reliance on family and community networks for accessing healthcare and measures of patient strategy when interacting with formal healthcare systems. This section also included measures of use of traditional healers and self-rated assessments of the efficacy of traditional healers. The final section included measures of NCD prevalence and the use of lifelong medication.

The measures in the final section of the questionnaire, as well the measures on general and specific healthcare access were adapted from the Adult Questionnaire of the South African Demographic and Health Survey (3). The questionnaire was piloted in October of 2008, and the final questionnaire administered from November 2008 to June 2010. The questionnaire took less than an hour to complete.

#### Rural context: literature review

The rural context was provided by a literature review related to NCD prevalence and healthcare access within the Agincourt subdistrict. Most of the findings were provided by research reports based on a 2006 INDEPTH–World Health Organisation (WHO) study on global ageing and adult health as well as other quantitative research conducted within the Agincourt subdistrict.

### Conducting the case studies

The case studies employed a qualitative methodology incorporating serial narrative interviews to present an experience-based overview of concepts of disease causation, self-treatment, and coping. Data was collected in Soweto between October 2009 and February 2010, and in Agincourt between July and September 2010. Where possible, two interviews were conducted with each respondent in both study sites. Each interview was conducted in the home of the research participant. The primary questions were:

1. How do women experience chronic illness?; and
2. How do women experience healthcare systems (formal and informal) in relation to their chronic illness?

The sampling for the Soweto qualitative case study was based on the initial findings of the survey. At the midway point of data collection, a group of women was randomly selected within a stratified population of women who had one or more NCDs and who were willing to participate in a follow-up study. This population was dually stratified by intensity of disease (determined by number of NCDs and use of lifelong medication) and the use of support systems (home-based care or other support systems). Participants were selected within each stratum to ensure a broad range of experience, including mild and severe cases of chronic illness. All participants had lived with their condition for at least a year.

A different sampling strategy was employed in Agincourt. The research was conducted in three villages within the subdistrict, which were selected based on key informant discussions with resident researchers based in the MRC/Wits Rural Health and Health Transition Unit in Agincourt. In order to focus on the acceptability of healthcare services in Agincourt, the selection criteria were such that the villages selected would most closely resemble Soweto in terms of access to healthcare facilities and basic infrastructure. Thus all three villages had a local clinic, although distinctive rural features remained, such as the use of communal water taps.

The rural study participants were selected from a list of respondents within the three villages who had participated in the 2006 INDEPTH–WHO Study on Global Ageing and Adult Health, and who had indicated the presence of one or more long-term illnesses, including arthritis, stroke, angina, diabetes, chronic lung disease, asthma, depression, hypertension, cataracts, and loss of teeth. A purposive sampling technique was employed to ensure sufficient variation in terms of village of residence and number of reported NCDs. Selection was concluded once it became clear that the initial coding categories were exhausted.

### Quantitative data analysis: Soweto and Agincourt

The primary data (data collected by the 2008–2010 survey) were descriptively analysed using STATA/IC 10.0. A series of Pearson’s Chi-squared analyses described the basic relationships between demographic and disease state characteristics and healthcare-seeking behaviour. The analysis included multivariate logistic regressions to show the adjusted effects of individual, societal, and healthcare system factors on healthcare utilisation.

### Qualitative data analysis: Soweto and Agincourt

In each study site, data collection and analysis occurred concurrently, with each process informing the other, and data collection proceeding until a ‘saturation point’ had been reached (referring to the exhaustion of conceptual categories for identifying key observations) (15). The serial methodology enabled the development of initial coding schemes during the process of data collection, which were later presented to the research participants during the second interview.

After data collection, a content–analytic approach was conducted for the additional analysis of textual data. While the interview schedule provided a broad guide for formulating the analysis plan, additional layers of subthemes were inductively derived through the raw data (15). During this phase, the transcripts were hand coded (coded without recourse to software) using an open coding technique to sort through the data and develop additional coding categories. A dual process of data analysis was followed to sort through the interview transcripts:

1. Listening to voices within interviews: including the compilation of fieldnotes, inductive identification of themes, and the compilation of interview summaries; and
2. Locating themes across interviews: including comparative analyses across all the interview transcripts conducted in the study site and the further development of the coding scheme and the collation of interview sections by theme.

An independent scientist not connected with the study followed the same inductive coding process with a sample of the transcripts and we found a high level of agreement in our coding. At this point, the data analyses were presented in separate research papers based on the qualitative site study conducted in each site.

### Ethics

Each participant gave her informed consent by signature to participate in the study and to have the interviews recorded and the recordings kept by the researcher for a period of 6 years. The Human Research Ethics Committee (Medical) of the University of the Witwatersrand (M090235) approved the research. All personal information was kept confidential, and study participants are referred to by pseudonyms.

## Results

### The urban context

#### Historical trends in the use of formal and informal healthcare in Soweto

From 1990 to 1992, 1994, 1995, and from 2002 to 2006, caregivers reported on place of treatment for a range of childhood illnesses. While we should be cautious in inferring from these data trends of healthcare utilisation for NCDs, the data may be taken as broadly indicative of general healthcare utilisation trends in Soweto–Johannesburg from the early 1990s to around 2006.

**Fig. 1:**
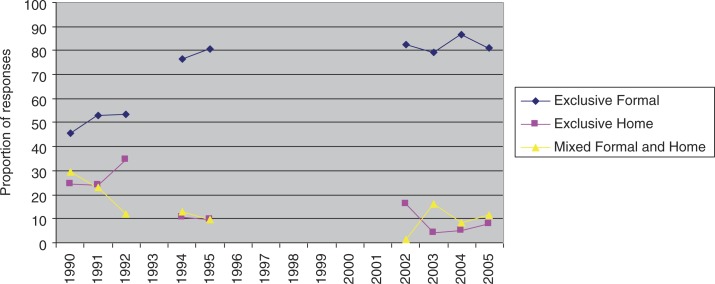
Overall use of formal and informal healthcare from 6 months to 16 years.

The data available for the analysis occurs in two phases: from 1990 to 1995, and from 2002 to 2006, with a large gap in the data from 1996 to 2001. Despite the gap in the data, a trend of increasing exclusive use of formal healthcare services and a decreasing trend of exclusively informal and mixed formal and informal healthcare utilisation is suggested by the cross-sectional analysis (Fig. 1).

Although such findings should be interpreted with caution, the picture which emerges regarding broad healthcare utilisation trends in Soweto is one of increasing reliance on formal healthcare systems, represented by clinics, hospitals, and private doctors, and the decreasing use of informal healthcare systems, represented by home remedies and traditional medicine.

### Healthcare access and belief in Soweto: The 2008–2010 survey

The aim of this component of the study was to describe the current healthcare access, beliefs, and practices of middle-aged and old women residing in Soweto.

#### Study demographics

A total of 1,102 women participated in the survey. The demographic profile of the survey sample describes a low resource population with a high prevalence of NCDs.

Over one-third (37.3%) of the population could be categorised as having a low socio-economic status, defined as access to only one or less of five socio-economic items, including indoor running hot and cold water, indoor flush toilets, living in a house, owning a house, and owning a motor vehicle (Table 1).

**Table 1:** Population characteristics of Bt20 caregivers between 2008 and 2010 (*N*=1,102)

Variable	Category	*N*	%
Age	30–39	266	24.1
	40–49	589	53.5
	50–65	245	22.2
	Missing	2	0.2
	Total	1,102	100
Socio-economic indicators (ownership)	Indoor hot and cold water (*n*=1,102)	405	36.8
	Flush toilet inside (*n*=1,102)	538	48.8
	Live in house (*n*=1,101)	980	88.9
	Own house (*n*=1,101)	885	80.3
	Own motor vehicle (*n*=1,099)	348	31.6
	High SES (4–5 items)	190	17.2
	Medium SES (2–3 items)	496	45.0
	Low SES (0–1 item)	411	37.3
	Missing	5	0.5
	Total	1,102	100
Employment	Formal or informal paid labour	582	52.8
	Housewife/pensioner/unemployed	519	47.1
	Missing	1	0.1
	Total	1,102	100
Self-reported chronic non-communicable disease	No chronic disease	543	49.3
	One chronic disease	339	30.8
	More than one chronic disease	219	19.9
	Missing	1	0.1
	Total	1,102	100

Slightly over half the respondents (50.7%) reported having at least one NCD. Of this group, 32.5% had high blood pressure, 12.8% had arthritis, 8.7% had high blood cholesterol, and 6.5% had diabetes. The remaining 17.4% of responses were distributed over a variety of NCDs, which individually included less than 5% of the total responses. Of those reporting some form of NCD, over two-thirds (69%) used prescribed lifelong medication on a daily or regular basis.

#### Access to healthcare services

Access to healthcare services was determined by measures of availability and affordability. The availability of healthcare services was determined by whether a healthcare service provider was available within a 2 km radius or 20 min walking distance from the respondent. Those who reported availability of healthcare service providers in their neighbourhood were asked whether they felt the services were affordable for them.

Private medical practices were more easily available than government clinics (75.1 and 61.5%, respectively), although not as affordable (59.1 and 83.6%, respectively) (Table 2). While sangomas (traditional healers) were easily available for almost a third of the respondents (32.1%), they were reported as the least affordable (28.2%) of the formal and informal healthcare services.

**Table 2:** Access to healthcare services in Soweto as reported by Bt20 caregivers between 2008 and 2010 (*N*=1,102)

	Proportion of respondents reporting availability within 2 km radius (%)	Proportion of respondents reporting affordability (if available) (%)
Private medical practice (*n*=1,102)	75.1	59.1
Private hospital or clinic (*n*=1,102)	7.4	52.4
Government or community clinic (*n*=1,102)	61.5	83.6
Government hospital (*n*=1,102)	3.9	88.4
Community organisation (*n*=1,102)	30	63.8
Pharmacist (*n*=1,101)	37	72.9
Sangoma or traditional healer (*n*=1,101)	32.1	28.2
Herbalist (*n*=1,101)	15	63.9
Faith healer or priest (*n*=1,102)	18.2	57.5

#### Experiences of healthcare services

In the context of a high prevalence of NCDs in the study population, it is surprising that slightly less than a quarter of the respondents (24.3%) reported an illness within the past 6 months which obliged them to access a healthcare service. The precise phrasing of the question was: ‘Have you had any illness or condition in the last 6 months so that you have had to seek treatment or healthcare?’ Those with NCDs were significantly more likely to report such illnesses (*p*<0.05), with around a third (33.3%) actually doing so.

We asked all respondents (*n*=1,102) what were the main problems they experienced in accessing healthcare from government clinics and hospitals. The top five problems (mentioned by over 75% of respondents) were long waiting times (24.5%), unfriendly staff or poor service (17.8%), lack of medication (17.3%), overcrowding (13.9%), and short consultation times (5%). Around 12% of respondents felt that there were no problems in accessing public healthcare services.

#### Social characteristics and healthcare access

In order to explain the apparently low healthcare utilisation of participants with NCDs, we examined the relationships existing between social characteristics and disease status (Table 3), focusing particularly on those respondents with NCDs (excluding from the analysis respondents with TB).

**Table 3:** Correlating healthcare utilisation of persons with NCDs with individual, societal, and healthcare system determinants in the Bt20 cohort 2008–2010 (*N*=1,089)

		Utilisation	No utilisation	Total	*P*
Individual determinants	Age (mean±SD) (*N*=545)	46±6.7	45.1±6.5	45.7±6.6	*p*=0.07
	Socio-economic status (*N*=542)				
	Low	34 (18.9%)	51 (14.1%)	85 (15.7%)	*p*=0.15
	Medium to High	146 (81.1%)	311 (85.9%)	457 (84.3%)	
	Employment (*N*=545)				
	Formal or informal paid labour	81 (44.8%)	144 (39.6%)	225 (41.3%)	*p*=0.25
	Housewife/pensioner/unemployed	100 (55.3%)	220 (60.4%)	320 (58.7%)	
	Medical aid (*N*=545)	41 (22.7%)	52 (14.3%)	93 (17.1%)	*p*=0.01
	Regular medication (*N*=545)	126 (69.6%)	253 (69.5%)	379 (69.5%)	*p*=0.98
Societal determinants	Social support for participants with chronic disease				
	Shared health beliefs family (*N*=545)	179 (98.9%)	361 (99.2%)	540 (99.1%)	*p*=0.75
	Shared health beliefs community (*N*=545)	142 (78.5%)	302 (83%)	444 (81.5%)	*p*=0.2
	Specific belief in traditional healers (*N*=542)	66 (36.9%)	108 (29.8%)	174 (32.1%)	*p*=0.09
	Use of patient strategies (*N*=545)	61 (33.7%)	89 (24.5%)	150 (27.5%)	*p*=0.02
Healthcare system	Availability of formal healthcare services (*N*=545)	165 (91.2%)	306 (84.1%)	471 (86.4%)	*p*=0.02
	Affordability of formal healthcare services (*N*=545)	136 (75.1%)	244 (67%)	380 (69.7%)	*p*=0.05

A means comparison test suggests that the age of women with an NCD was closely, although not significantly, related to healthcare utilisation (*p*=0.07) (Table 3). Pearson’s Chi-square tests indicate that the possession of medical aid was the strongest individual level factor influencing the utilisation of healthcare (*p*=0.01). Amongst societal level factors, the self-reported use of patient strategies was significantly related to the utilisation of healthcare services.

The use of patient strategies was determined by the combination of two statistically associated Likert-scale measures regarding the selective disclosure of the symptoms of illness, and the private rehearsal of what symptoms to present to formal healthcare workers. Those respondents with NCDs who reported using patient strategies were significantly more likely to use healthcare services (*p*=0.02).

Specific belief in traditional healers, or the belief that there were some conditions which could only be treated by traditional healers, and not by doctors, was closely, although not significantly related to healthcare utilisation (*p*=0.09). These beliefs were positively associated with healthcare utilisation. Both availability and affordability of healthcare services were significantly positively related to reported healthcare utilisation (*p*=0.02 and *p*=0.05).

Multivariate logistic regression of individual, societal, and healthcare system factors influencing the utilisation of healthcare services in the past 6 months by persons with NCDs confirms the importance of the possession of medical aid for using healthcare services (OR=1.7, CI=1.01–2.84). It also confirms the close relationship between healthcare utilisation and the use of patient strategies for negotiating healthcare access (OR=1.6, CI=1.04–2.34).

### The rural context

At the time of data collection, infrastructure within the Agincourt subdistrict was limited, although undergoing rapid development. The area was marked by an absence of formal sanitation, erratic supply of piped water to communal standpipes, gravel roads, and limited electricity supply. Local employment and farming was limited, with most adults seeking work elsewhere. There was one health centre and five satellite clinics within the site, and three public hospitals within 60 km of the site (13).

#### Health status in Agincourt

The INDEPTH–WHO study conducted in Agincourt suggests that the prevalence of NCDs in Agincourt is comparable to that in Soweto, with 42% of the study participants reporting muscular-skeletal pain and 31% reporting hypertension. Around 43% of participants who did not report hypertension had high blood pressure levels compatible with hypertension (16). Elsewhere, hypertension has been reported as the commonest risk factor in Agincourt, with 43% of the population having some degree of hypertension (17).

The INDEPTH–WHO study reports that around 80% of participants who had hypertension reported taking medication for their condition in the past 12 months, but also shows similar trends with the Soweto data in that 43% of the sample, a third of whom had been diagnosed with a NCD, reported not needing any healthcare in the past 12 months (16). This may be due to the presence of multiple disease narratives. For example, Thorogood et al. (17) have found that hypertension is understood both naturalistically and socially in Agincourt, resulting in a variety of treatments commensurate with the differing causes. However, Thorogood et al. (17) point out that the majority of people with high blood pressure (around 76%) were not taking any treatment due to obstacles originating at the clinic.

### The qualitative case studies

#### A brief description of the study participants

For the qualitative site studies, a total of 22 interviews were conducted with 12 participants in Soweto, and a total of 25 interviews were conducted with 13 participants in Agincourt.

There was a difference in the age ranges in both study sites (Table 4). The participants in Soweto were younger, with an age range of 45–91. Ten of the 12 respondents were under 60 years of age. In Agincourt the age of participants ranged from 55 to 90, with only one of the participants being under the age of 60. The discrepancy in age across both study sites was due to the different nature of the sampling frames. In Soweto, the sampling frame consisted of the Bt20 caregivers, the biological mothers of children born in 1990. In Agincourt, the sampling frame was specifically fixed at individuals over the age of 50.

**Table 4:** Quantitative comparison of case study participants in Soweto (*N*=12) and Agincourt (*N*=13), 2009–2010

	Soweto (*N*=12)	Agincourt (*N*=13)
Age (mean)	54.9	70.9
Age (range)	45–91	55–90
Hypertension	7 (58.3%)	11 (84.6%)
Muscular-skeletal pain	7 (58.3%)	6 (46.2%)
Diabetes	4 (33.3%)	4 (30.8%)
Other	7 (58.3%)	4 (30.8%)
Comorbiity	9 (75%)	10 (76.9%)
Allopathic treatment	12 (100%)	12 (92.3%)
Alternative treatment	6 (50%)	8 (61.5%)

There was no great difference in the range of NCDs of the research participants in both sites. Quantitatively, the two groups were similar in terms of their acceptance of formal healthcare services. All of the participants in Soweto, and all but one of the participants in Agincourt primarily used formal healthcare services to cope with their condition. Eight participants in Agincourt and six in Soweto reported using some form of complementary medicine.

### The urban case study (Soweto)

#### Experiencing chronic illness

Due to the presence of study participants with severe cases of chronic illness in Soweto, the implications of chronic illness were more dwelt upon, as in the case of Thandi, aged 49, who had arthritis. During the interview she stated that at one time she underestimated her disease: ‘When you are young you don’t see it’s a problem’ (Thandi, 49, Soweto). However, this view changed with the development and experience of arthritis. She went on to say:

When you are locked in your bones, in your knees, you can’t walk. When your spine is shifted, you can’t move. You can’t do anything. So the pain in your body is so painful, the pain in your body. It is so painful that you can’t sleep, you can’t do anything, and there are no tablets which can help you. (Thandi, 49, Soweto)

In this particular case, Thandi reported using a combination of healthcare approaches to manage her illness. She initially consulted a private doctor, and was advised that she had arthritis of the spine. She was advised to go for an operation, but refused, and subsequently treated her condition by wearing a special corset recommended by the same doctor. She said of the tablets recommended to her by the doctor, ‘it works just to minimize the pain. It’s not something that is healing’ (Thandi, 49, Soweto). Rather, she concentrated her treatment on the taking of a cinnamon and honey mixture, which was recommended to her by a friend. She said of this mixture, contrasting it with the tablets prescribed by her doctor: ‘It heals. It is not going to harm me’ (Thandi, 49, Soweto). She attributed to this mixture her remarkable recovery from the initial symptoms of arthritis: ‘Now I am walking, I am doing everything’ (Thandi, 49, Soweto).

#### Complex aetiologies

Problems within the family, such as disease, death, and unemployment were spoken of as causes of chronic illness. Chronic illness was related to crises brought about by the accumulated strains of life. Two respondents ascribed their condition to the strains of looking after their grandchildren, in one instance due to the death of the parent. Sarafina traced her arthritis to the time just after she married, when she was living with her husband’s family, and, to relieve the stress, she would do the washing:

I used to wash a lot with my hands. I was taking out the stress. I was living with my in-laws by then, so I was taking out the stress with my washing. (Sarafina, 45, Soweto)

Sarafina was a married and self-employed business woman who baked and sold confectionary. Although she initially said that her arthritis was not very severe, during the course of the interview it became clear that her condition was at one time fairly severe, and still had an impact on her life. On the advice of her husband, she consulted a private doctor in the neighbourhood who prescribed some tablets which she shortly afterwards discontinued. Her greatest help in coping with arthritis she felt were the black rubber rings and bracelets which she wore on her hands. She explained:

I was in the [minibus] taxi and there was an elder guy at my left side. I was taking money from the back. Yabona, every time, when I lift my hand it will ‘Qa’, it will make a sound ‘Qa! Qa! Qa!’, and that gentleman said to me … ‘You know this bracelet from the paraffin drums?’ I said ‘Yes’, ‘You must use them. Wear those bracelets, I am telling you, your arthritis will be fine’ …. He told me that my bones are fragile and what. And since then they helped me a lot. (Sarafina, 45, Soweto)

#### Experiencing healthcare

The clinic is the first point of entry in the overall healthcare system, offering a range of free basic services at the community level. In Soweto, the visit had become a familiar routine, taking place about once a month. Respondents described a dual procedure at the clinic: one for dispensing prescribed medication, and the other for diagnosis and treatment. The process for dispensing medicine is, on the surface, a quick and efficient one: the visit to the clinic is entered into a personal file and a prescription is issued. The process is called ‘repeats’:

Now at the clinic they have got, they call it ‘repeats’. When you go there they just view your card, they put the stamp, and they sign for which room to go. You don’t queue, you just go to fetch your medication, and they don’t help you, they don’t take your blood pressure and your sugar. (Rosaline, 49, Soweto)

#### Medicine-taking

In Soweto, we found a preoccupation with how the medicine from the clinic interacts with the body. Shirley articulated her ambivalence as concern about what the pills were doing to her body:

But when I’m alone I ask myself ‘I take these tablets everyday, and then, what are they doing to my body?’ and then you know what I do? I drink a lot of water. I think I’m washing them out. (Shirley, 53, Soweto)

Rosaline relates her experience as follows:

Once I was washing dishes, so one tablet … fell on the floor. So I said ‘OK, I’ll take another one’. So I take that pill and throw it in the sink. I close the tap, and wash the dishes. I finish, take out the water and, I thought, you know it would melt, and it didn’t, and from there I get worried. ‘What is happening? What is going on when I drink these tablets everyday in my system? Where does it ….?’ I don’t know where it is going. What is going on in my system? You know? You drink that pill everyday, and you know? And I sit down, and take that pill, and take two spoons and I try to press it, to squeeze it, to squeeze it. It never squeezes. You know? [starts to laugh]. (Rosaline, 49, Soweto)

Rosaline relied mainly on Chinese teas to regulate her high blood pressure, although she intermittently took the tablets from the clinic. Her goal was to wean herself off the clinic medication.

### The rural case study (Agincourt)

#### Experiencing chronic illness

In Agincourt respondents described a variety of symptoms of chronic illness (‘high blood’, ‘dizziness’, ‘headaches’, ‘not having power’, ‘sugar diabetes’, and ‘stress’), but in many cases they tended to understate the severity of their illness. For example, respondents answered the question ‘What is your condition?’ in various ways:

I am fine, it’s just that I was not feeling well. (Linah, 60, Agincourt)

I can say that I am not that much ill exactly. (Katherine, 63, Agincourt)

I consulted the doctor, but I was taking it easy. (Thabiso, 61, Agincourt)

None of the participants appeared particularly uncomfortable during the interviews. Their chronic illnesses imposed some inconvenience, but they were not in themselves major causes of distress. They were described as ‘just the illnesses of nowadays’ (Katherine, 63, Agincourt). Most respondents had resorted to using their pension money to employ people to help them farm and perform domestic duties, particularly washing.

### Complex aetiologies

Three broad explanations for the causes of chronic illness emerged from the interviews in Agincourt: 1) occupational causes: resulting from strenuous work; 2) dietary causes: related to increase of meats, oil, sugar, and processed foods in diets; and 3) social causes: related to ‘thinking too much’ about family difficulties, such as the loss of children. Social causes were most prominent in the interviews, particularly as they related to stressful social circumstances, such as the stress of losing a family member or the stress of motherhood, as described by 63-year-old Katherine:

If you are a mother everything that is painful it will pass by you. (Katherine, 63, Agincourt)

In Agincourt two respondents were caring for their grandchildren due to the death of their children. These respondents, who both had high blood pressure, attributed their illness to the stress of looking after their grandchildren:

I’m thinking too much because of my grandchildren. You find that I don’t have anything to help them. I do get the pension, but I can’t get by with that only, so I’m always thinking. That is why I know that the cause of the high blood is because I’m thinking too much. (Marta, 82, Agincourt)

The death of their children brought added pain to the stresses of life, which can be felt in the reflection: ‘if you have lost your children things are difficult. I have some, but I am not satisfied’ (Phipas, 63, Agincourt).

#### Experiencing healthcare

Respondents described a uniform experience at the clinic, characterised by minimal interaction with the clinic staff and patients, and centred around collecting medication. One respondent described the process as follows:

What they are doing at the clinic, they check the BP [blood pressure]. So they will tell you if it is high or low. If they find that it is high they will give you the small pill. After a few minutes they will recheck, and if they find that it is still high they will give you the same pill. Then you go home. (Idah, 63, Agincourt)

A unique feature of formal healthcare access in Agincourt was the purposeful use of clinics or doctors further away. The theme of ‘better services elsewhere’ emerged in the interviews, such as with Katherine, aged 63, who never used the local health centre in Agincourt, preferring to use Xanthia clinic further away. In this instance, the preference was guided by a low opinion of the confidentiality and caring-nature of the nurses at the health centre. Hunadi avoided using her local clinic for counseling services because of low staffing, and saved money for transport to clinics further away. Nomses, aged 70, could afford private doctors, and therefore she avoided clinics and the long queues found there. But even for private doctors, she preferred travelling to Hazyview, some 60 km away.

### Medicine-taking

In Agincourt, a common and powerful response to the question: ‘What helps you most to cope with your illness?’ is simply to go to the hospital or clinic to collect pills. For Linah, the clinic is where she has ‘gotten life’ because of the clear diagnosis and treatment (Linah, 60, Agincourt). Marta, who relied on the clinic treatment, contrasted it with home and traditional remedies in terms of the relative simplicity of the clinic treatment:

I don’t use any home remedy. I don’t even know how to mix it. Even if you can explain to me how they mix I will forget, because I’m not used to it. What I know is the medicine from the clinic …. I’m not educated but I know how to take the pills and how they are working. (Marta, 82, Agincourt)

Marta was very clear about relying only on the clinical medication, saying ‘if you concentrate on the pills you will live’ (Marta, 82, Agincourt). However, Marta’s preference for clinic-based medicines was closely connected with religious-based concepts of maintaining good health, as is evident in the following statement:

Even if you just drink medicine, if you don’t believe you won’t get well. So the only thing is to believe in what you are doing. (Marta, 82, Agincourt)

Church membership, particularly of African Christian Churches, was the strongest factor motivating against the open use of traditional medicine, although the cost of traditional healers was also mentioned as a reason for not using them.

Cabiya, aged 89, while consulting a number of doctors for her sore stomach, also tried a traditional healer. She tried the medicine, but it did not work, and so she discontinued the treatment. She then started attending a Pentecostal church, and therefore she now ‘cannot go’ to the traditional healer (Cabiya, 89). As a member of the congregation, she is expected to abstain from consulting traditional healers. The church advises her to consult only the clinic, and to take only the medicines prescribed by the clinic. However Cabiya’s church encourages the use of a particular alternative remedy, in this case, Vaseline, which ‘removes white sputum’ (Cabiya, 89).

In Agincourt only Thabiso and Phipas were confident in discussing home remedies. Phipas, aged 63, suffered from high blood pressure, and supplemented her clinical treatment with a home remedy: a local herb called *nkanka*. The herb is sometimes used as a dietary supplement with porridge, but, when boiled, can be drunk as a tea for high blood pressure. Phipas sometimes voluntarily abstained from the pharmaceutical medication, which made her feel dizzy, relying solely on *nkanka* when she felt her heart beating fast.

## Discussion

Soweto and Agincourt share similar patterns of healthcare utilisation and healthcare beliefs. Only a small portion of individuals in both study sites with NCDs used formal healthcare services. In Soweto, where over half (50.7%) of the participants of the quantitative survey reported having some form of NCD, only a third (33.3%) of these participants reported accessing healthcare in the past 6 months. In Agincourt, studies show that not only may hypertension be under-reported (16), but at least a third of people with NCDs do not access healthcare for ongoing treatment (16, 17). These low healthcare utilisation rates are comparable to that reported in the South African Demographic and Health survey, which reported a public healthcare utilisation rate of 24% for adult women (3). These contextual findings are mirrored more broadly in the regional context, with one systematic review suggesting that in sub-Saharan Africa up to 60% of people with hypertension have not been diagnosed, and up to 70% of those who have been diagnosed are not regularly taking treatment (18).

The development of a lifeworld/healthworld schema potentially addresses the concerns of low healthcare utilisation by focusing our attention on lifeworld rationalisation. In this process public health researchers and social scientists prioritise body narratives within formal healthcare institutions (9). In such a research enterprise, the motivation is not to promote a particular type of healthcare utilisation but to explore and publicise the lifeworld/healthworld of participants in healthcare processes.

A close consideration of the qualitative case studies suggests at least five themes within the lifeworld/healthworld which should feature in any comprehensive schema:

1. Processes of constructing body narratives, focusing on the need of individuals with chronic illness to seek healthcare experiences which enhance the construction of narratives.
2. Encounters with purposive–rational systems, characterised by varying degrees of sociological observation regarding the experience of the formal healthcare system. In Agincourt, we are confronted with the unexpected finding, at least in terms of previous research conducted in Agincourt (17, 19) and other African contexts (20) that, for older women with chronic illness, clinical treatment and pharmaceutical medication is a prominent feature of treatment, reinforced by church-based healthcare practices.
3. Encounters with traditional medicine: While traditional medicine appeared irrelevant in the Soweto case study, it appeared more taboo in Agincourt. However, the quantitative survey in Soweto suggests that, like in Agincourt, traditional healers may be regularly consulted for non-biomedical purposes. None of the respondents in the quantitative survey reported using traditional healers or herbalists to treat diseases, a finding reported elsewhere in South Africa (3, 21). However, almost a third of respondents in Soweto (31.8%) felt that there were some diseases which could only be treated by traditional healers.
4. Encounters with contemporary informal medicine: The use of contemporary forms of informal medicine, which featured particularly strongly in the Soweto case study, but which was also not entirely absent from the Agincourt case study is indicative of a transforming social environment marked by a modernised view of healthcare in terms of multiple market-related commodities. At the same time, the scope such views provide for individualised approaches to coping with chronic illness are also related to the need for establishing a communicatively rationalised experience of chronic illness. Such observations are possible even in highly modernised contexts, where similar trends in the use of holistic and individualised treatments for chronic illness have been found (22).
5. Religion and healthcare, which featured more strongly in the rural case study, is a prominent theme with multiple research possibilities focussing on the role played by religious profession in relationship with traditional norms and taboos. In our findings, religious belief played an important role in delineating the proper uses of traditional medicine, and generally excluded such usage from the domain of healthcare practice.

### Mapping the lifeworld/healthworld schema

**Fig. 2:**
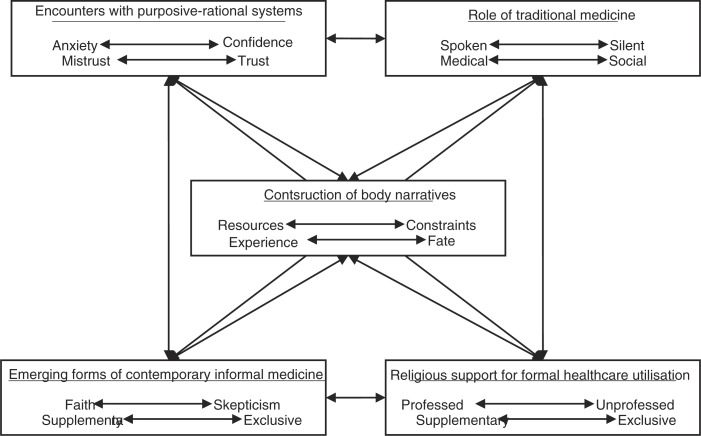
Conceptual map of the lifeworld/healthworld schema.

Each component of the lifeworld may be conceived as consisting of a series of polarities that describe communicative practice between the individual and social institutions related to healthcare. For example, narratives describing encounters with purposive–rational systems may tend towards constructing the self as a passive recipient subjected to anxiety or towards the construction of the self as an active and confident participant in purposive–rational systems. Similarly the role of traditional medicine is described by narratives which tend to make hidden the role of traditional healers, and those which make it explicit, and by narratives which describe a medical or social role for traditional healers (Fig. 2).

### Implications for healthcare policy and practice

The development of a broader interest in the use of body narratives to illustrate the lifeworld/healthworld of persons with NCDs has the potential to inform emergent healthcare approaches which aim to bridge hermeneutic and objectivest public healthcare. This ‘third way’ has been described as the domain of healthcare practitioners who integrate evidence-based medicine with narrative approaches (6). Indeed, a narrative approach to the formation of treatment plans has long been a *de facto* practice in a number of institutional settings. In Africa community-based organisations, particularly faith-based organisations (FBOs) have used these approaches in providing healthcare services (23). Often competing with FBOs in this regard in many African countries are traditional healers, who generally embody accepted world views and who rely on narrative technique for diagnosis and treatment (24).

In the South African context, body narratives and lifeworld/healthworld rationalisation have a potentially revolutionary role to play in current attempts to revitalise PHC. For example, the use of body narratives and lifeworld/healthworld rationalisation are particularly enhanced by the participation of community healthworkers and community-based organisations, both of which play a key role in the development of community-oriented primary care (COPC). In this regard, a coherent lifeworld/healthworld schema will prove integral in the primary aims of COPC not only in ensuring the congruence of formal healthcare services with community needs but also in designing and implementing community-based interventions based on in-depth assessments of community healthcare needs (25).

### Study limitations

We have discussed how our sampling strategies in both Agincourt and Soweto were determined by the available data in both study sites. This resulted in an older group of participants in Agincourt, who generally had milder forms of chronic illness than the Soweto group. This may partly explain the predominance of themes of anxiety and mistrust of formalised healthcare in Soweto, yet our thematic analysis also made it clear that social mores, etiquette, and custom exert a greater influence over the healthcare encounter in Agincourt. This may suggest the possibility of social desirability bias in the findings, particularly in relation to the expressed approval of the clinic and disavowal of traditional medicine. At the same time, we note the consistency of the narratives which explain different reasons for the preference of clinical medication, including the simplicity of the treatment.

We were limited in the use we could make of contextual data for both qualitative site studies. Whereas in Soweto we had access to a wealth of primary and secondary data for contextualising the urban case study, in Agincourt we had to make do with a review of previous studies carried out in that subdistrict. This limitation is partly addressed by the fact that the study is exploratory, and greater emphasis is placed on the qualitative site studies than in the contextual findings. Even given this limitation, it is clear that Agincourt and Soweto display similar broad trends of healthcare utilisation and health beliefs.

## Conclusion

The problem statement for this study focused on how to construct meaningful research findings within the context of several contrasting dichotomies, both in terms of discourses of NCDs (or chronic illness) and approaches to healthcare access. We sought to integrate these dichotomies through exploring the lifeworld/healthworld of women with NCDs in an urban and rural area. Our aim was to explore how women with NCDs experience their illness and access healthcare in an urban and rural area, and our objectives were to formulate historical–comparative community descriptions of the study sites and to analyse the experiences of chronic illness and healthcare in both study sites.

Our first objective was addressed by an analysis of primary and secondary data for Soweto, and a review of previous research conducted in Agincourt. We found that both sites show similar trends of low utilisation of formal healthcare services, a high prevalence of NCDs, as well as the presence of multiple healthcare beliefs.

Our second objective was addressed by thematic content analysis of a number of serial in-depth interviews conducted in both study sites. The results of this analysis highlighted a concern not with the availability, affordability, or acceptability of formal healthcare services, or even an under utilisation of those services, but an overwhelming concern with the development of body narratives – of meaningful and personal ways of talking about and coping with chronic illness.

In our discussion of the findings, we argue that the diverse concerns raised by the findings can be met by the adoption of an integrated healthcare approach focused on lifeworld/healthworld rationalisation and the ongoing development of body narratives. It is to be hoped that the current thesis marks the first step in realising this vision.

## References

[1] Mayosi BM, Flisher AJ, Lalloo UG, Sitas F, Tollman SM, Bradshaw D (2009). The burden of non-communicable diseases in South Africa. Lancet.

[2] Goudge J, Gilson L, Russel S, Gumede T, Mills A (2009). Affordability, availability and acceptability barriers to healthcare for the chronically ill: longitudinal case studies from South Africa. BMC Health Serv Res.

[3] Department of Health, Medical Research Council, OrcMacro (2007). South Africa Demographic and Health Survey 2003.

[4] Coovadia H, Jewkes R, Barron P, Sanders D, McIntyre D (2009). The health and health system of South Africa: historical roots of current public health challenges. Lancet.

[5] Kautzky K, Tollman SM, Barron P, Roma-Reardon J (2008). A perspective on primary health care in South Africa. South African health review 2008.

[6] Martin CM, Peterson C (2008). The social construction of chronicity – a key to understanding chronic care transformations. J Eval Clin Pract.

[7] Kleinman A (1988). Response: on reading Schweder’s reading of social origins of distress and disease. Cult Med Psychiatr.

[8] Conrad P, Barker K (2010). The social construction of illness: key insights and policy implications. J Health Soc Behav.

[9] Scambler G, Scambler G, Higgs P (2000). Medical sociology and modernity: reflections on the public sphere and the roles of intellectuals and social critics. Modernity, medicine and health: medical sociology towards 2000.

[10] Habermas J (1987). The theory of communicative action. Lifeworld and system: a critique of functionalist reason.

[11] Germond P, Cochrane JR (2010). Healthworlds: conceptualizing landscapes of health and healing. Sociology.

[12] Richter L, Norris SA, Pettifor J, Yach D, Cameron N (2007). Cohort profile: Mandela’s children: the 1990 birth to twenty study in South Africa. Int J Epidemiol.

[13] Kahn K, Tollman SM, Collinson MA, Clark SJ, Twine R, Clark BD (2007). Research into health, population and social transitions in rural South Africa: data and methods of the Agincourt health and demographic surveillance system. Scand J Public Health Suppl.

[14] Robbins D (1997). Agincourt: a district health demonstration site.

[15] Zhang Y, Wildemuth BM, Wildemuth B (2009). Qualitative analysis of content. Applications of social research methods to questions in information and library science.

[16] Gomez-Olive F, Thorogood M, Clark B, Khan K, Tollman S (2013). Self-reported health and health care use in an ageing population in the Agincourt sub-district of rural South Africa. Glob Health Action.

[17] Thorogood M, Connor MD, Lewando Hundt G, Tollman SM (2007). Understanding and managing hypertension in an African sub-district: a multidisciplinary approach. Scand J Public Health Suppl.

[18] Addo J, Smeeth L, Leon DA (2007). Hypertension in sub-saharan Africa: a systematic review. Hypertension.

[19] Goudge J, Gumede T, Gilson L, Russel S, Tollman SM, Mills A (2007). Short communication: coping with the cost burdens of illness: combining qualitative and quantitative methods in longitudinal, household research. Scand J Public Health Suppl.

[20] Hjelm K, Mufunda E (2010). Zimbabwean diabetics’ beliefs about health and illness: an interview study. BMC Int Health Hum Rights.

[21] Nxumalo N, Alaba O, Harris B, Chersich M, Goudge J (2011). Utilization of traditional healers in South Africa and costs to patients: findings from a national household survey. J Public Health Policy.

[22] Brien S, Bishop FL, Riggs K, Stevenson D, Freire V, Lewith GT (2011). Integrated medicine and the management of chronic illness: a qualitative study. Br J Gen Pract.

[23] Green A, Shaw J, Dimmock F, Conn C (2002). A shared mission? Changing relationships between government and church health services in Africa. Int J Health Plann Manage.

[24] Madamombe I Traditional healers boost primary health care: reaching patients missed by modern medicine. Africa Renewal 2006.

[25] De Maeseneer J, Flinkenflogel M (2010). Primary health care in Africa: do family physicians fit in?. Br J Gen Pract.

